# Necessary Conditions for Reliable Propagation of Slowly Time-Varying Firing Rate

**DOI:** 10.3389/fncom.2020.00064

**Published:** 2020-07-29

**Authors:** Navid Hasanzadeh, Mohammadreza Rezaei, Sayan Faraz, Milos R. Popovic, Milad Lankarany

**Affiliations:** ^1^Clinical and Computational Neuroscience, Krembil Research Institute, University Health Network, Toronto, ON, Canada; ^2^School of Electrical and Computer Engineering, College of Engineering, University of Tehran, Tehran, Iran; ^3^KITE Research Institute, Toronto Rehabilitation Institute, University Health Network, Toronto, ON, Canada; ^4^Institute of Biomedical Engineering, University of Toronto, Toronto, ON, Canada

**Keywords:** time-varying rate coding, information propagation, feed-forward neural network, noise, network size, synaptic delays

## Abstract

Reliable propagation of slow-modulations of the firing rate across multiple layers of a feedforward network (FFN) has proven difficult to capture in spiking neural models. In this paper, we explore necessary conditions for reliable and stable propagation of time-varying asynchronous spikes whose instantaneous rate of changes—in fairly short time windows [20–100] msec—represents information of slow fluctuations of the stimulus. Specifically, we study the effect of network size, level of background synaptic noise, and the variability of synaptic delays in an FFN with all-to-all connectivity. We show that network size and the level of background synaptic noise, together with the strength of synapses, are substantial factors enabling the propagation of asynchronous spikes in deep layers of an FFN. In contrast, the variability of synaptic delays has a minor effect on signal propagation.

## Introduction

Information in the brain is encoded by either the number of spikes in a relatively long time window, i.e., rate code, or by their precise timing, i.e., temporal code (Abeles et al., 1994; Panzeri et al., 2001, 2017; Montemurro et al., 2007; Kremkow et al., 2010; London et al., 2010; Zuo et al., 2015; Runyan et al., 2017; Noble, 2019). The feasibility of utilizing both coding strategies has also been shown in different neural systems (Kumar et al., 2010; Lankarany et al., 2019). In temporal coding, information is carried by groups of neurons that fire *synchronously*, as in synfire chains (Abeles et al., 1994; Diesmann et al., 1999), whereas in rate coding, neuronal firing ideally remains asynchronous across neurons (Litvak et al., 2003). Information processing in a hierarchically organized cortical system relies on the reliable propagation of synchronous and asynchronous spikes (Joglekar et al., 2018).

The reliable propagation of synchronous spikes (temporal code) is well-understood and relatively easy to implement in computer models (Kumar et al., 2008, 2010; Joglekar et al., 2018). In contrast, the reliable propagation of rate-modulated asynchronous spiking (rate code) is poorly understood and remains challenging to implement in computer models (Litvak et al., 2003). Indeed, spikes may synchronize as the signal progresses through deeper layers or may tend toward an attractor state representing quiescence or a fixed rate. In all of the scenarios, rate-based coding is compromised.

Several studies have addressed the conditions required for spike-rate propagation (Shadlen and Newsome, 1994; van Rossum et al., 2002; Litvak et al., 2003; Wang et al., 2006; Kumar et al., 2008, 2010; Joglekar et al., 2018; Barral et al., 2019; Han et al., 2019). Shadlen and Newsome (Shadlen and Newsome, 1994) demonstrated the feasibility of rate transmission using leaky integrate and fire (LIF) models receiving balanced excitatory and inhibitory inputs. Nevertheless, Litvak et al. (2003) showed, using the same parameters quoted by Shadlen and Newsome (1994), that rate was not reliably transmitted when >2 layers were considered. They concluded that rate transmission in FFNs is highly unlikely. van Rossum et al. (2002) showed the feasibility of reliable transmission of instantaneous firing rate (asynchronous spikes) in un-balanced FFNs where the input to each layer is delivered as an injected current. Kumar et al. (2008) studied conditions for propagating synchronous and asynchronous spikes utilizing biologically realistic network models. The coexistence of firing rate and synchrony propagation was shown under precise combinations of synaptic strength and connection probability (Kumar et al., 2010). Cortes and van Vreeswijk (2015) showed that the pulvinar thalamic nucleus allows for asynchronous spike propagation through the cortex; they supply the input-output firing rate relationship between two cortical areas without manipulating synaptic strengths.

Neuronal networks with feedforward connections consisting of excitatory neurons are thought of as the model for linking upstream neurons with downstream neurons, either across different layers within the same cortical region or between different cortical regions. Recent studies have capitalized on the role of recurrent connections in reliable transmission of synchronous and asynchronous spikes (Joglekar et al., 2018; Barral et al., 2019). A recent study has demonstrated that networks with recurrent excitation and lateral inhibition stabilize signal transmission (Joglekar et al., 2018). As well, a recurrent network including AMPA and NMDA-mediated components that operates in an excitatory-inhibitory balanced regime (with respect to both magnitude and time of excitatory and inhibitory synaptic inputs) has been recently proposed as a novel model of information propagation (Barral et al., 2019).

Despite undoubtedly significant impacts of biologically realistic network architectures in conveying information across/between layers, neuronal networks with feedforward connections play a substantial role in understanding the mechanisms of faithful propagation of different types of spikes (Kumar et al., 2010). Due to the simplicity of feedforward networks (FFNs) compared to networks with recurrent connections, the impact of different factors like intrinsic properties of neurons in reliable propagation of spikes can be better studied using FFNs. For example, Han et al. (2019) showed that layer-to-layer heterogeneity arising from lamina-specific cellular properties facilitates propagation of synchronous and asynchronous spikes in FFNs.

In this paper, we focus to systematically explore the necessary conditions underlying which information of time-varying asynchronous spikes can be reliably transmitted in deep layers of an FFN. Using optimal synaptic weights (Faraz et al., 2020), we study the roles of (i) network size, (ii) the level of background synaptic noise, and (iii) the variability of synaptic delays in propagation of slowly time-varying asynchronous spikes. We show that unlike the variability of synaptic delays that has a minor effect on signal propagation, network size and the level of background synaptic noise are substantial factors enabling the propagation of asynchronous spikes in deep layers of an FFN.

## Methods

Network size, level of background synaptic noise, and variability of synaptic delays are the main factors that were investigated in a feedforward architecture. We created an FFN composed of excitatory neurons, modeled by leaky integrate and fire (LIF) model, receiving shared input from the previous layer plus background synaptic noise. We calculated coding fraction—representing goodness of propagation of time-varying asynchronous spikes—for different levels of background synaptic noise, network size, and variability of synaptic delays.

### Optimal vs. Fixed Synaptic Weights

To test the effect of each factor, we estimated synaptic weights using a reduced network model whose function, in the propagation of a shared input, is equivalent to an FFN with all-to-all connectivity [see (Lankarany, 2019; Faraz et al., 2020) for more details]. Schematic representations of the reduced network and an FFN with all-to-all connectivity were shown in Figures S1A,B. To better distinguish between the performances of an FFN with and without optimal synaptic weight, we calculated the input signal of the second layer of an FFN given the spikes of the first layer. This input, the reconstructed stimulus, was shown in Figures S1C,D for optimal and fixed synaptic weights, respectively. The value of the fixed synaptic weights generated 0.5 mV postsynaptic potential (per spike), which is within a biologically realistic range. As can be seen in these figures, the stimulus reconstructed by optimal synaptic weights significantly better tracked the original stimulus (specifically for the optimal range of network size and noise level). For a reduced network model with a fixed number of neurons and a constant level of synaptic noise, the optimal weights were estimated to minimize the L2-norm error between the reconstructed (in the first layer) and original stimulus (Faraz et al., 2020). Of note in the reduced model, is the vector representation of the synaptic weights (rather than the matrix form) that enables the use of convex optimization techniques to calculate these weights. Given the estimated weights in the reduced network model, the mean and standard deviation (std) of the weights were calculated. To obtain synaptic weights for an FFN with all-to-all connectivity, we drew samples from a Gaussian distribution of the mean and std of the estimated weights. Codes for estimating synaptic weights are available at https://github.com/nsbspl/async-spike-propagation.

### Slow Stimulus

The slow signal was delivered to the neurons in the first layer of an FFN and modeled by an Ornstein-Uhlenbeck (OU) process of the time constant of 50 msec. This slow signal can, for example, represent the luminance of a natural stimulus (Lankarany et al., 2019). The OU process can be written as:

(1) $$\begin{array}{r} \frac{dx}{dt}=-\frac{x\left(t\right)-\mu }{\tau }+a\sqrt{\frac{2}{\tau }}\;\xi \left(t\right) \end{array}$$

where ξ is a random number drawn from a Gaussian distribution with 0 average and unit variance. τ is the time constant, μ and α indicate the mean and standard deviation of variable *x*, respectively. The mean and variance of the slow signal is 16 and 15 pA, respectively.

### Level of Background Synaptic Noise (OU Process)

Background synaptic noise was modeled by an OU process of the time constant of 5 msec (Destexhe et al., 2001). The mean of synaptic noise is 0 that balances the effect of background excitatory and inhibitory synaptic inputs. The standard deviation of noise varied in a range of (5, 10, 15, 20, 25, 30, 35, 40) pA, which generated background (no stimulus) spiking activity in a range of (1.2–10.4) Hz.

### Neuron Model

We used leaky integrate and fire (LIF) model. The dynamics of membrane potential is expressed as follows.

(2) $$\begin{array}{r} \frac{dV}{dt}=\frac{-\left(V-E_{L}\right)+RI_{inj}}{\tau_{V}} \end{array}$$

where *E*_L_ = −70 mV, *R* = 1 MΩ, and τ_*V*_ = 10 msec. *I*_*inj*_ indicates the injected current (slow signal as the stimulus of the first layer). Spike occurs when V≥Vth, where *V*_*th*_ = −40 mV and the reset voltage is −90 mV. For the neurons in the subsequent layers, *I*_*inj*_ is equal to the total presynaptic input (weighted by synaptic strengths) plus independent synaptic noise. A double exponential function of τ_*rise*_ = 0.5 *msec* & τ_*fall*_ = 5 *msec* was used to model an identical synaptic waveform.

### Network Size

Our computational study was performed by varying the number of neurons in an FFN. Network sizes were varied in a range of [50, 100, 200, 300, 400, 500, 750, 1000].

### Variability in Synaptic Delays

Variability in synaptic delays introduced heterogeneities in the network. This variability can be interpreted as an un-equal distance between pre- and post-synaptic neurons (see Discussion). We modeled such variability by a Gaussian distribution whose mean represents the synaptic delay (3 msec) and standard deviation indicates the level of variability across neurons. The std of synaptic delays varies within an interval of (0:0.25:1.5) msec, which is in agreement with small variations of synaptic latencies in the cortex (Boudkkazi et al., 2007).

### Instantaneous Firing Rate and Optimal Kernel Width

The instantaneous firing rate was calculated by convolving superimposed spikes (of each layer of an FFN) with a Gaussian kernel. To achieve a consistent comparison between the firing rates across layers, we used a Gaussian kernel of width = 25 msec. This kernel width was near to optimal for spikes in the first layer of the FFN (regardless the network size and noise level). We used a method proposed in Shimazaki and Shinomoto (2010) to calculate the optimum (Gaussian) kernel width of the spikes in the first layer. The optimal kernel width was equal to 21.8 msec. We plotted the instantaneous firing rates estimated by these widths against each other in Figure S2 to demonstrate the similarities between these estimates.

### Coding Fraction and Kullback–Leibler Divergence

To quantify how much network size and the level of background synaptic noise effect the propagation of asynchronous spikes in a network, we used coding fraction (CF) that represents how good the instantaneous firing rate of the second layer tracks that of the first layer. CF is calculated as follows.

(3) $$\begin{array}{r} CF=1-\frac{||Firing\left(Layer\;2\right)-Firing\left(Layer\;1\right)|{|}_{2}}{||Firing\left(Layer\;1\right)|{|}_{2}} \end{array}$$

where *Firing (Layer 1)* and *Firing (Layer 2)* denote the instantaneous firing rate of the 1st and the 2nd layers, respectively. And, ||.||_2_ indicates the norm 2. CF lies within [−1, 1], where 1 represents perfect transmission.

Besides, in order to validate whether CF is equivalent to an information-theoretic measure, we calculated Kullback–Leibler (KL) divergence measures between the instantaneous firing rate of the second and the first layers of the FFN. The Kullback–Leibler (KL) divergence (Timme and Lapish, 2018) quantifies the similarity between the probability distributions of the instantaneous firing rates of the first (*Fr*_1_) and the second (*Fr*_2_) layers. The KL divergence is defined as (Perez-Cruz, 2008):

(4) $$\begin{array}{r} D_{KL}\left(P_{Fr_{2}}\left(r_{2}\right),P_{Fr_{1}}\left(r_{1}\right)\right)=\sum_{r_{1}\in Fr_{1},\;r_{2}\in Fr_{2}}P_{Fr_{2}}\left(r_{2}\right)*\\ \;\log\left(\;\frac{P_{Fr_{2}}\left(r_{2}\right)}{P_{Fr_{1}}\left(r_{1}\right)}\right) \end{array}$$

where *P*_*F*_*r*__1__ and *P*_*F*_*r*__2__ are the probability distributions of the firing rates, namely, *Fr*_1_ and *Fr*_2_, respectively. To calculate these probability distributions, we utilized a non-parametric estimation method to approximate them using normal kernel smoothing (Bowman and Azzalini, 1997). The estimated density function, ${\hat{f}}_{h}\left(x\right)$, for each layer, can be written as follows.

(5) $$\begin{array}{r} {\hat{f}}_{h}\left(x\right)=\frac{1}{Nh}\overset{N}{\underset{i=1}{\sum }}K\left(\frac{x-x_{i}}{h}\right)\;;\;-\infty <x<\infty \end{array}$$

where *N* is the sample size, *K*(.) is the kernel function, and *h* is the bandwidth. *D*_*KL*_ is non-negative (≥ 0) and non-symmetric in *P*(*r*_2_) and *P*(*r*_1_). *D*_*KL*_ is equal to zero if *P*(*r*_2_) and *P*(*r*_1_) match exactly, and can potentially equal infinity if there is no similarity between the two distributions. In other words, the KL measure is equal to zero if the firing rates of both layers have the same statistical characteristics, implying a perfect information propagation (across two layers).

We calculated CF and KL divergence for different levels of background synaptic noise (for *N* = 400 and *N* = 500). Figure S3 shows that these two measures are inversely correlated, i.e., a higher CF is equivalent to a lower KL divergence, which represents more similarities between the probability distributions of the firing rates, and thus, a better propagation of the asynchronous firing rates.

### Synchrony Measure

Similar to Lankarany et al. (2019), we used a threshold method to detect synchronous events. Synchrony threshold, for each layer of an FFN with specific network size, noise level, and optimal synaptic weights, was chosen such that the superimposed spikes during a 10 msec interval generate >20 mV post-synaptic potential. As the optimal synaptic weights depend on the network size and noise level, the synchrony threshold can be interpreted as a minimum number of spikes required for post-synaptic neurons to fire in a short interval.

## Results

### Network Size and Level of Background Synaptic Noise Are Substanital Factors That Enable Reliable Propagation of Slowly Time-Varying Asynchronous Spikes in FFNs

To explore the effects of network size and the level of background synaptic noise in the propagation of slowly time-varying asynchronous spikes, we vary these parameters in an FFN consisting of two layers and calculate the coding fraction (CF). In addition, the number of synchronous events (see Methods) is quantified to provide more numerical characterizations of the roles of the above parameters in signal propagation.

A recent study (Barral et al., 2019) has shown that channel capacity and decoding accuracy decreased after the first layer but remained above the chance (and almost unchanged). However, the rationale behind using two layers is that the propagation of information across layers of an FFN is more likely to maintain robust if the information is preserved within the first two layers.

**Figure 1:**
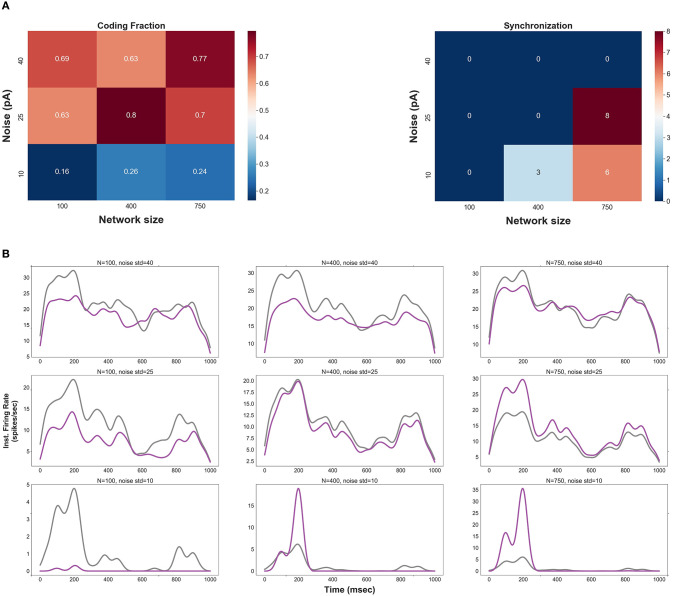
**(A)** Coding fraction (CF), CF is plotted for different values of network size and the level of background synaptic noise (in a two layers FFN). **(B)** Instantaneous firing rate of the 2nd layer (purple) vs. that of the 1st layer (gray) for different pairs of network size and noise level. Note that the delay between layers is compensated in these curves.

Figure 1A shows CF and synchrony measure of spikes as a function of network size and std of the background synaptic noise. Three values underlying each parameter are chosen to classify the range of network size and level of noise into three categories, namely, small (low), medium (moderate), and large (high). Note that spikes in the second layer of an FFN (for each pair of parameters) are produced by optimal synaptic weights (see Methods and Distribution of Optimal Synaptic Weights Depends on Network Size and Level of Synaptic Noise).

CF increases for medium and large network sizes with moderate to high levels of background noise. However, CF is relatively low outside these ranges; it decreases for small network sizes regardless of the noise level. More precise characterization of CF against different values of network size and noise level (see Figure S4) indicates that CF has the highest values for a specific range of parameters, i.e., network sizes in a range of {400–500} and std of the noise in a range of {20–30}^pA^.

The number of synchronous events increases for medium- and large-size FFNs with low levels of noise (also note that the signal is not well represented in small-size FFNs with low noise). Thus, one can predict that more synchronous events would be generated in the subsequent layers for low levels of background synaptic noise. Figure 1A (right) also shows that synchronous events occur in large-size FFNs with moderate level of noise, suggesting that an improper ratio of network size and noise level causes synchrony propagation in the subsequent layers.

To visually inspect the effects of network size and noise std, the instantaneous firing rates of the first and the second layers are shown in Figure 1B. CF is relatively high for (i) large network sizes (≥750) with a high level of synaptic noise (σ = (35–40) pA) and (ii) small network sizes (<300) with a high level of synaptic noise (σ = 40 pA). In (i), signal propagation in deeper layers is not stable due to the high level of noise: the rate code remains asynchronous but tends to the average firing rate (see Figure S5B). As will be discussed in section Distribution of Optimal Synaptic Weights Depends on Network Size and Level of Synaptic Noise, the corresponding synaptic weights are very weak and biologically unrealistic (*N* = 750, σ = 40 pA). The high level of noise in (ii) makes the network spontaneously active, so it is not possible to discern any signal from background synaptic activity. As shown in Figure 1B, a large network size (*N* = 750) with a small level of background activity (σ = 10 pA) increases the shared input activity and amplifies the signal in the second layer. In this case, synchrony increases across the layers (see also Figure S5C for medium-size network with low noise). In medium-size FFNs (*N* = 400), small (10 pA) and large (40 pA) synaptic noise amplifies and attenuates signal propagation, respectively.

**Figure 2:**
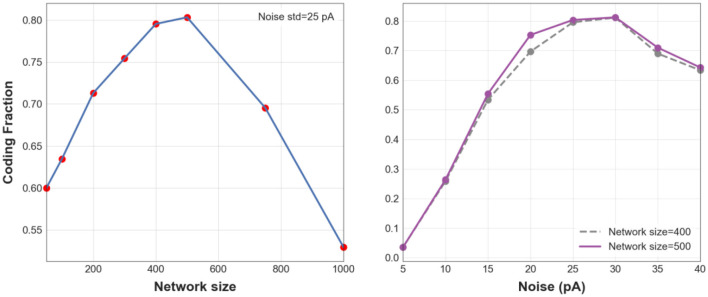
1-D CF. CF vs. different network sizes when noise std is equal to 30 pA (**Left**). CF vs. std of synaptic noise (**Right**) for FFNs with 400 (blue dashed line) and 500 neurons (purple).

To better understand the effects of network size and noise level on the transmission of asynchronous spikes within the first two layers of an FFN, CF is plotted in Figure 2 for different values of each factor while the other factor is kept constant. Figure 2 (Left) shows CF for different network sizes when the noise std, σ = 30 pA. CF is maximized for *N* = 400 and *N* = 500, meaning that information of asynchronous spikes is best transmitted to the 2nd layer of an FFN for particular network sizes (given a fixed level of synaptic noise). Similarly, Figure 2 (Right) shows CF as a function of background synaptic noise for FFNs consisting of 400 and 500 neurons. CF decreases for small and large (CF = 0.56 for σ = 50 pA, data is not shown in the figure) levels of background synaptic noise which implies that information is best transmitted to the 2nd layer of a medium-size FFN (*N* = 400, 500) given a moderate level of synaptic noise, i.e., σ = 30 pA.

**Figure 3:**
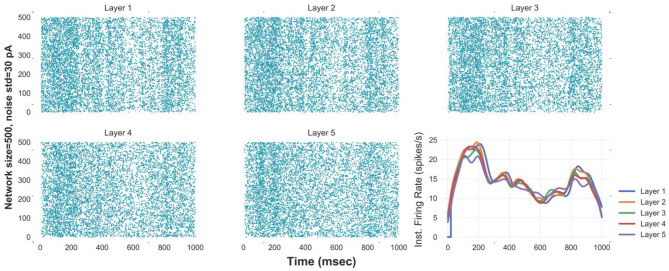
Reliable propagation of slowly-time varying asynchronous spikes in 5 layers [raster plot & instantaneous firing rate (Gaussian kernel of width 25 msec)] of FFNs with optimal values of network size (500), level of synaptic noise (30 pA), and synaptic strength. Note: the delay between layers is compensated in these curves.

Simulating an FFN with the optimal network size and level of synaptic noise enhances the propagation of asynchronous spikes in deeper layers. To evaluate the performance of an FFN with the optimal parameters in the propagation of asynchronous spikes in deeper layers, we show the raster plot and the instantaneous firing rate of an FFN up to five layers in Figure 3. Stable propagation of asynchronous spikes can be seen in this figure for *N* = 400, σ = 30 pA (as well as in Figure S5A). The instantaneous firing rates represent signal propagation across layers (bottom right curves in Figure 3). Previously, it was shown that a homogeneous random network could not transmit a completely asynchronous population activity due to the existence of the residual correlations (Kumar et al., 2008). Such correlations are mainly originated from the shared connectivity and could be reduced by increasing the network size (Kumar et al., 2008). Unlike homogeneous random networks, in an FFN with all-to-all connectivity, i.e., a dense network, a larger network size results in a larger number of shared pre-synaptic inputs. Although background synaptic noise induces some heterogeneities in the network, the propagation of asynchronous spikes in deeper layers is not enhanced for network sizes > 750. It is to be noted that increasing the noise level in large network sizes remains spikes propagated asynchronously; however, at the expense of losing information of time-varying firing rates (Figure S5B). Moreover, decreasing the level of background synaptic noise causes synchronous spikes built up in the subsequent layers of a medium-size FFN (Figure S5C).

### Distribution of Optimal Synaptic Weights Depends on Network Size and Level of Synaptic Noise

In addition to synaptic noise that provides heterogeneities in an FFN [or a locally connected random network (Mehring et al., 2003)], previous studies considered that a distribution of passive properties of neurons that rises to a distribution of the synaptic weights (Kumar et al., 2008) induces heterogeneities across neurons. In this study, synaptic weights are estimated from the firing activities of the first layer, and the underlying mean and standard deviation (with an assumption of Gaussian distribution) are used to draw weights for subsequent layers. Thus, the mean of the estimated weights reflects the strength of the synapses, and the std of the weights indicates heterogeneities across synapses.

**Figure 4:**
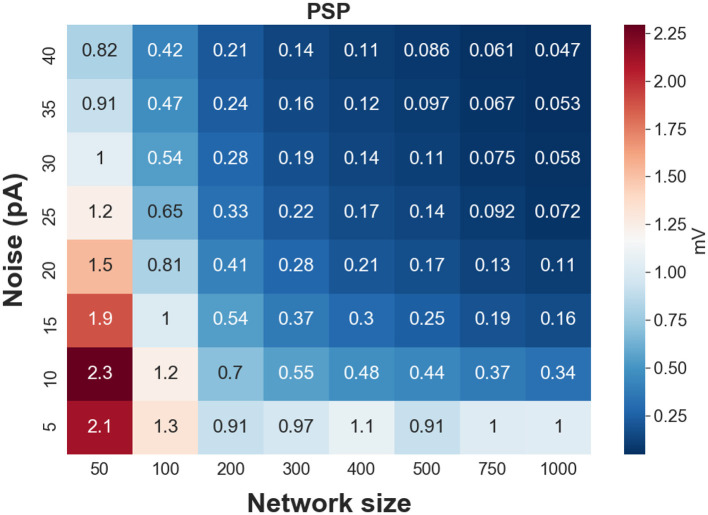
Heat map of postsynaptic potentials (corresponding to synaptic weights) for different network sizes and levels of synaptic noise.

To test whether the estimated weights are biologically realistic, we validate if the corresponding postsynaptic potential (PSP) lies within a biologically-reasonable range. We show in Figure 4, a heatmap of PSPs—corresponding to the mean of the estimated weights—as a function of network size and level of background synaptic noise. Synaptic weights generating extremely weak (<0.05 mV) or extremely strong (>1.5 mV) PSPs per presynaptic spike are considered as unrealistic weights. As can be seen in this figure, the synaptic weights for large network sizes (*N* > 750) and moderate to high background noise are weak, generating postsynaptic potentials less than 0.1 mV (per single presynaptic spikes). In contrast to the large network sizes with weak synapses, the estimated synaptic weights of FFNs with small network sizes generate larger postsynaptic potentials, specifically for the lower levels of synaptic noise. Therefore, one can conclude that only a specific range of synaptic weights is biologically realistic.

**Figure 5:**
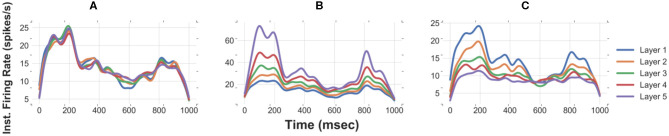
Improper synaptic weights result in either attenuation or amplification of the slow signal in the subsequent layers. Instantaneous firing rates of an FFN with *N* = 500 and noise std = 30 pA for **(A)** proper synaptic weights, **(B)** strong synapses (corresponding to *N* = 400 and noise std = 30 pA), and **(C)** weak synapses (corresponding to *N* = 600 and noise std = 30 pA).

Improper selection of synaptic weights for an FFN results in unstable propagation of asynchronous spikes. Systematically varying synaptic strengths from moderately weak to moderately strong synapses, we find a transition between attenuation mode, where transmission of time-varying rates failed, to an amplification mode where the average firing rate increased at the subsequent layers (Figure 5). We repeat running an FFN of size 500 with the level of noise of 30 pA with synaptic weights (mean and std) obtained from an FFN with the same level of synaptic noise but different network sizes, i.e., relatively stronger synapses (0.1 *pA/mV* corresponding to *N* = 400) and weaker synapses (<0.05 pA/mV corresponding to *N* = 600). Figure 5 demonstrates that the propagation of a slow signal is either amplified (Figure 5B) or attenuated (Figure 5C) when the weights are stronger or weaker, respectively.

It is to be noted that the time constants of excitatory synapses and neurons' membrane potential influence the estimated synaptic weights. Chan et al. (2016) showed that different combinations of these time constants alter the pair-wise correlation and synchrony of spikes, e.g., burst firing occurs when the time constant the neuron's membrane is small and that for excitatory synapses is large. These time constants are fixed in our estimation method (see Methods), and no burst firing was observed in medium-size FFNs (*N* = 400, 500) with a moderate level of synaptic noise (20, 25, and 30 pA).

### Variability of Synaptic Delays Has a Slight Influence on the Propagation of Asynchronous Spikes

**Figure 6:**
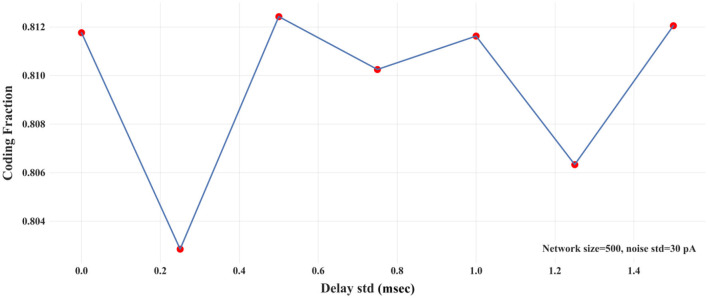
CF vs. std of synaptic delays in a two-layer FFN with *N* = 500 and noise std = 30 pA.

In biologically-realistic scenarios, presynaptic inputs are delivered from neurons with different distances to the target (postsynaptic) neuron. In the context of signal propagation, for example, Joglekar et al. (2018) have introduced inter-areal delays by considering the corresponding inter-areal wiring distances (Markov et al., 2014) and assuming a constant axonal conduction velocity. To explore how different distances between the pre- and post-synaptic neurons affect signal propagation, we introduce variability in the synaptic delays. Synaptic delays are drawn from a Gaussian distribution of a fixed mean (3 msec) and variable std. We calculate CF for different values of the std of synaptic delays in a two-layer FFN (*N* = 500 and σ = 30 pA). Figure 6 shows that CF is nearly independent of the variability of synaptic delays. In addition, we show the propagation of asynchronous spikes in an FFN with two levels of variabilities of synaptic delays in Figure S6. Similar to Figure 3, the FFN comprises 500 neurons, each receiving 30 pA synaptic noise. As expected, the variability of synaptic delays does not change signal propagation significantly compared to that with no variability.

### What Range of Firing Rates Can Be Reliably Tranmistted Across Multiple Layers?

**Figure 7:**
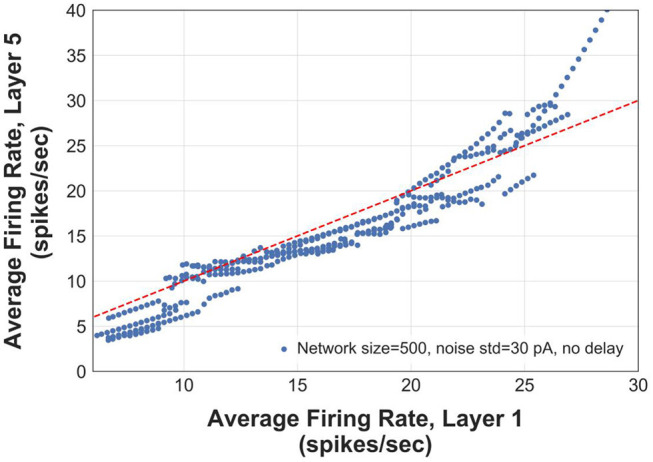
Logistic map showing output rate (firing rate in layer 5) vs. input rate (firing rate in layer 1). The red dashed line has a slope of 1.

We construct logistic maps of the firing rates in the first and the 5th layers of an FFN (*N* = 500 and σ = 30 pA) to determine the range of frequencies that can be reliably transmitted. Figure 7 shows that the information of asynchronous spikes is preserved across layers for frequencies in the range of [5–25] Hz. The curve in this figure is obtained by superimposing the average instantaneous firing rate of 5 networks, each of which receiving different slow stimuli (with the same time constants). For frequencies > 10 Hz, although the logistic curve becomes slightly sublinear, the firing rate of the 5th layer still tracks that of the first layer, indicating that the firing rate is uniquely represented in deep layers of the FFNs. However, for frequencies > 25 Hz, the logistic curve is supralinear that capitalizes on the tendency of the transmitted firing rates to the synchrony mode.

## Discussion

Necessary conditions for reliable propagation of time-varying asynchronous spikes in FFNs were investigated in this paper. Previous studies have addressed those conditions for transmission of synchronous spikes as well as the mean of firing rate of asynchronous spikes. However, these conditions barely remain valid for transmission of time-varying asynchronous spikes whose instantaneous rate of changes represents information of slow fluctuations of the stimulus. In this paper, we investigated the necessary conditions for reliable transmission of asynchronous spikes in an FFN. Specifically, we explored the effect of network size, level of background synaptic noise, and the variability of synaptic delays in an FFN with all-to-all connectivity. We demonstrated that network size and the level of background synaptic noise, together with optimal synaptic weights, were substantial factors that cooperatively enable the propagation of asynchronous spikes in deep layers of an FFN. Nevertheless, the variability of synaptic delays had a minor effect on signal propagation. Varying these factors systematically, we found that an FFN of network size of {400–500} with the level of synaptic noise in a range of {20–30} pA and proper synaptic weights transmits time-varying asynchronous spikes reliably.

Reliable propagation of a rate code is challenging: even weak pairwise correlations in the spike timing can notably deteriorate the fidelity of the rate code (Kumar et al., 2010). Thus, the robust transmission of asynchronous spikes cannot be simply achieved from a general theory based on the balance of (common) signal and (background) noise. We showed that the same level of (moderate) noise provides very different signal propagations in networks with different numbers of neurons; it degrades the firing rate of an FFN of size = 100 whereas it can maintain a reliable signal propagation for a network size of 400 (the synaptic weights are optimized in both networks). One can interpret that the network size and noise level are competing to maintain a constant signal to noise ratio. Nevertheless, this interpretation is not necessarily correct in the context of signal propagation through an FFN with all-to-all connectivity. For example, the performance of signal representation in the **second layer** of an FFN with *N* = 400 & σ = 25 pA is almost equivalent with that of *N* = 750 & σ = 40 pA (see Figure 1B). However, despite the similarity within the two layers, the firing rate of the latter degrades in the subsequent layers. Therefore, a specific range of parameters—network size, synaptic strength, and level of synaptic noise in an FFN—should be identified to cooperatively maintain a consistent representation of the common (slow) signal across multiple layers.

### Signal Propoagation in Neural Networks With Recurrent Connections

Despite the focus of this paper on the use of networks with feed-forward connections, recent studies demonstrated the feasibility of reliable signal propagation in recurrent networks (Joglekar et al., 2018; Barral et al., 2019). Of note in these studies is that lateral or feedback connectivity are biologically plausible architectures to transmit information between cortical layers (Stroud and Vogels, 2018). Joglekar et al. (2018) showed that reliable signal propagation could be achieved in large-scale recurrent network models of the macaque cortex. It is worth mentioning that the activity dynamics of recurrent networks can be compatible with those of FFNs (Kumar et al., 2010). Kumar et al. (2010) used FFNs as a part of a recurrent network to study reliable signal propagation in a biologically plausible scenario. In the context of visual perception, a recent study showed that a recurrent neural network with a few layers can be unfolded as very deep FFNs (Liao and Poggio, 2016; Rajaei et al., 2019). In addition, other studies illustrated that recurrent random networks may behave similar to an FFN (Ganguli et al., 2008; Goldman, 2009; Murphy and Miller, 2009; Kumar et al., 2010).

Thus, studying conditions of reliable signal propagation in an FFN helps better understanding the underlying mechanisms of information propagation in more biologically realistic scenarios in which the dynamics of an FFN interact with that of the embedding recurrent network.

### Impact of Heterogeneous Synaptic Delays on Signal Transmission in Recurrent Neural Network

We showed that the impact of heterogeneous synaptic delays on the propagation of asynchronous spikes in an FFN was not significant. However, the heterogeneity in synaptic delays can alter the dynamics of a recurrent neural network and have a substantial effect on signal transmission. Here, we discuss three scenarios that capitalize on the substantial but ambiguous impact of the variability of synaptic delays in signal propagation. First, the heterogeneity in synaptic delays might have contrasting effects if applied to excitatory and inhibitory neurons. In a strongly recurrent network with excitatory synapses, this heterogeneity can reduce the likelihood of synchronization. In contrast, it can alter the tight balance of excitatory and inhibitory inputs—necessary for a reliable signal propagation—in a recurrent network with lateral inhibition (Joglekar et al., 2018; Stroud and Vogels, 2018). Second, the variability of synaptic delays might be compromised by that caused by synaptic transmissions. A recent study demonstrated that a network with non-instantaneous synaptic transmission and fixed spike delivery delay is equivalent to a network with a proper distribution of spike delays and instantaneous synaptic transmission (Mattia et al., 2019). Thus, a network with various types of synapses is differently influenced by the heterogeneity of synaptic delays. For example, the impact of the variability of synaptic delays (e.g., std ~ 1 msec) can be more significant in a recurrent network with AMPA receptors (with a short time constant) compared to that with NMDA receptors (with a large time constant). Third, the variability of synaptic delays can be regulated by short- and long-term forms of synaptic plasticity. It was experimentally demonstrated that the synaptic latency at monosynaptically connected pairs of L5 and CA3 pyramidal neurons is inversely correlated with the amplitude of the postsynaptic current and sensitive to manipulations of the presynaptic release probability (Boudkkazi et al., 2007). Therefore, incorporating models of synaptic plasticity in the simulation of neural networks can induce another source of heterogeneity in synaptic delays, which in turn, alters signal propagation.

### Optimal Level of Synaptic Noise and Stochastic Resonance

Stochastic resonance in the neural systems was first observed in the response of the neuronal network to a weak periodic signal (Gluckman et al., 1996) [see (Bulsara et al., 1991; Longtin, 1993) for stochastic resonance in neuron models]. In stochastic resonance, a proper amount of noise can boost signal representation. Noise in our model recreated the effect of the background synaptic noise that exists *in vivo* (Destexhe et al., 2001). Previous studies, e.g., (van Rossum et al., 2002; Kumar et al., 2010), demonstrated that background synaptic noise may balance the synchronizing effect of shared connectivity in an FFN, thereby enabling the propagation of asynchronous firing rates. Unlike other studies that the average firing rate of asynchronous spikes is the sole information that must remain unchanged across layers, we used a time-varying slow signal as an input to an FFN to explore to what extent information of slow fluctuations can be transmitted across layers. In this study, although the input was not fully periodic, we found that a certain range of noise (see Results) enables maximum information transfer across layers. Interestingly, this range of noise (std of membrane fluctuations (no stimulus) ≈ 4 mV for std of noise = 25 pA) is in agreement with the level of background synaptic noise observed *in-vivo* high-conductance state (Destexhe et al., 2003). Thus, from a system level perspective and in the context of consistent information transfer, one can conclude that stochastic resonance might occur in an FFN with the optimal level of background synaptic noise (see also the effect of network topologies on stochastic resonance in FFNs Zhao et al., 2018).

### Optimal Network Size and System-Size Coherent Resonance

Similar to noise-induced resonance, an optimal number of elements in a biological system can maximize the regularity in the emitted signal (in the presence of optimal level of noise), i.e., system-size coherence resonance (Toral et al., 2003). Toral et al. (2003) demonstrated that there is a coherence resonance effect, in the sense of maximum regularity in the signal generated by an ensemble of globally coupled Fitzhugh-Nagumo models, as a function of the number of coupled neurons, namely, N, in the presence of noise. It was shown that, for a specific set of parameters, the maximum regularity occurs for N ≈160 (Toral et al., 2003). A coherence resonance with respect to the number of neurons may exist in an FFN. Both the stochastic resonance and coherence resonance (CR) were investigated in the triple-neuron feed-forward-loop network motifs (Guo and Li, 2009). It was demonstrated that noise could enrich the stochastic dynamics of those motifs. In this study, we showed that for specific distribution of synaptic strength and optimal level of background synaptic noise, a reliable propagation of the time-varying rate of asynchronous spikes occurs for medium size FFNs (in the rang of {400–500}). Despite differences in the types of inputs, the architecture of an FFN vs. excitatory/inhibitory coupled neurons, propagation vs. representation, and other factors that resulted in two different values underlying optimum network size [compared to Toral et al. (2003)], our study capitalized on the significance of system-size coherence resonance in neuronal dynamics.

## Data Availability Statement

The datasets generated for this study are available on request to the corresponding author. Codes are available at https://github.com/nsbspl/Necessary-Conditions-for-Reliable-Propagation.

## Author Contributions

NH: Conceptualization and analysis, programming, and writing the manuscript. MR and SF: Analysis and programming and contributing in the writing of the revised manuscript. MP: Supervising the study and revising the manuscript. ML: Conceptualization and analysis, design the study, programming, and writing the manuscript.

## Conflict of Interest

The authors declare that the research was conducted in the absence of any commercial or financial relationships that could be construed as a potential conflict of interest.
