# Supplementary material for: Necessary Conditions for Reliable Propagation of Slowly Time-Varying Firing Rate
Source: Front Comput Neurosci. 2020 Jul 29;14:64. doi: 10.3389/fncom.2020.00064 (PMC7405925; doi:10.3389/fncom.2020.00064)
Supplement: Supplementary file 1 [file Data_Sheet_1.docx]

**Figures (Supplementary)**

**(A) Reduced network model**

**(B) FFN with all-to-all connectivity**

**(C) Reconstructed stimulus using optimal synaptic weights**


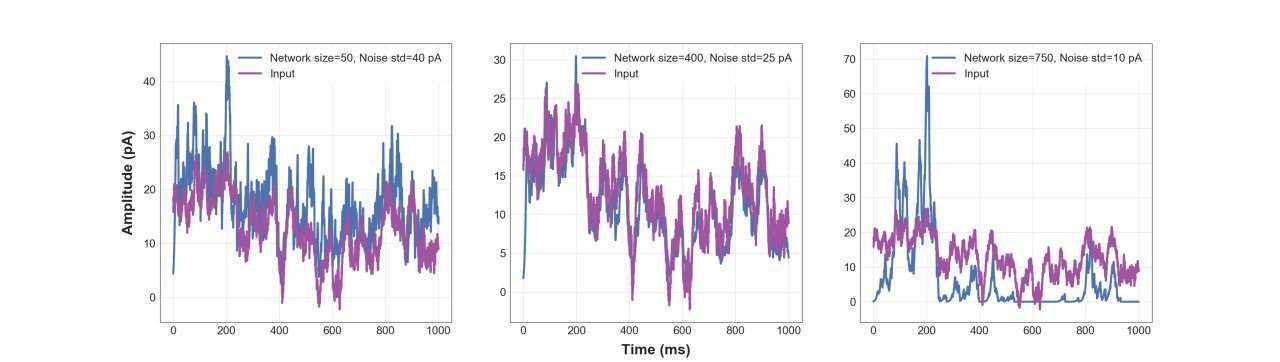


**(D) Reconstructed stimulus using fixed synaptic weights**


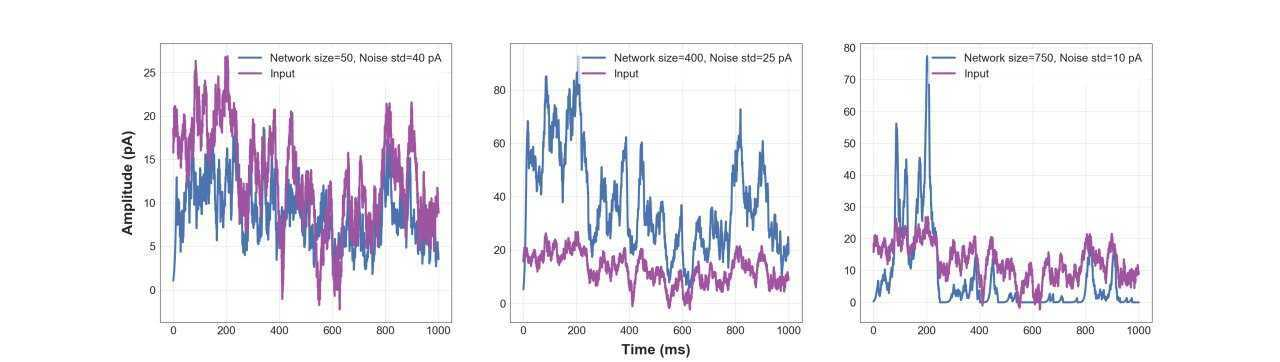


**Figure S1.** Schematic representation of the reduced network model (A) and an FFN with all-to-all connectivity (B). Reconstructed (blue) vs original (purple) stimulus using optimal synaptic weights (C) and fixed weights (D). Reconstructed stimulus, for each FFN with different network sizes and noise levels, was obtained by filtering (through synaptic functions) the spikes generated in the first layer. In fact, the reconstructed stimulus demonstrates the average input to the second layer of an FFN.


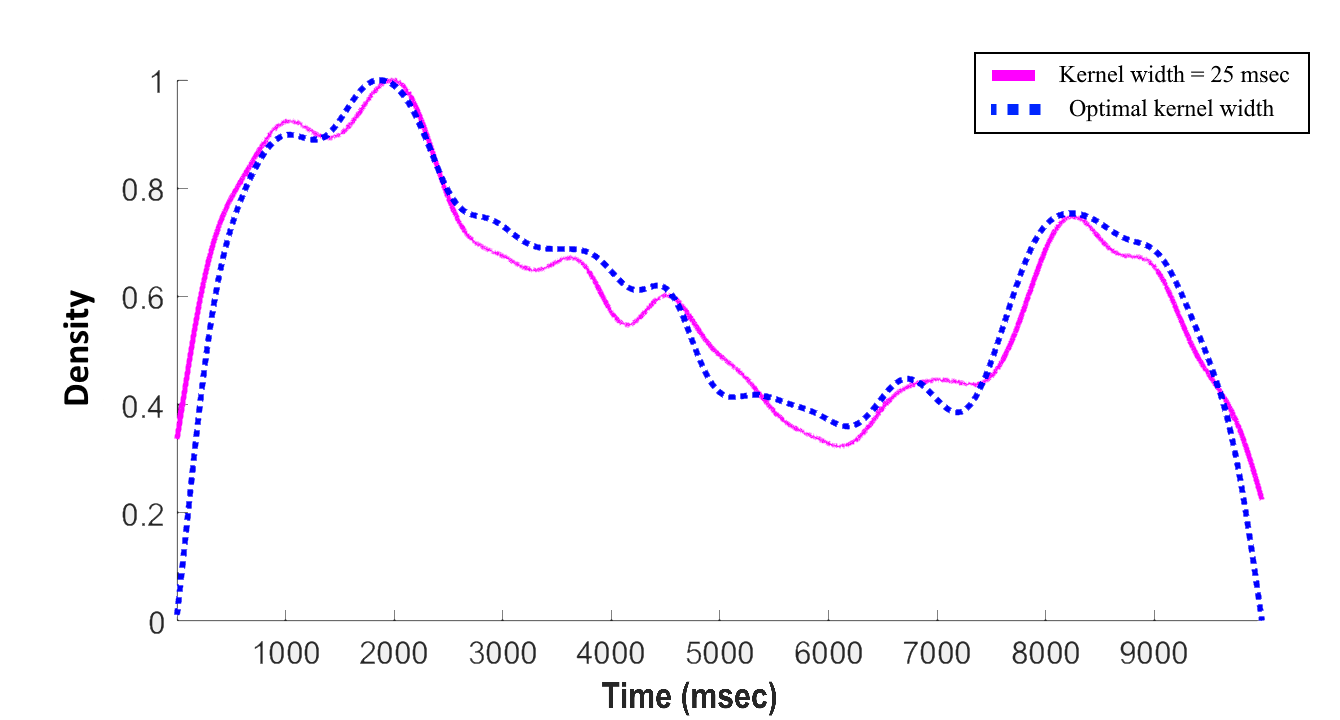


**Figure S2**. Instantaneous firing rate calculated by optimal Gaussian kernel width (black) vs. that obtained by 25 msec.


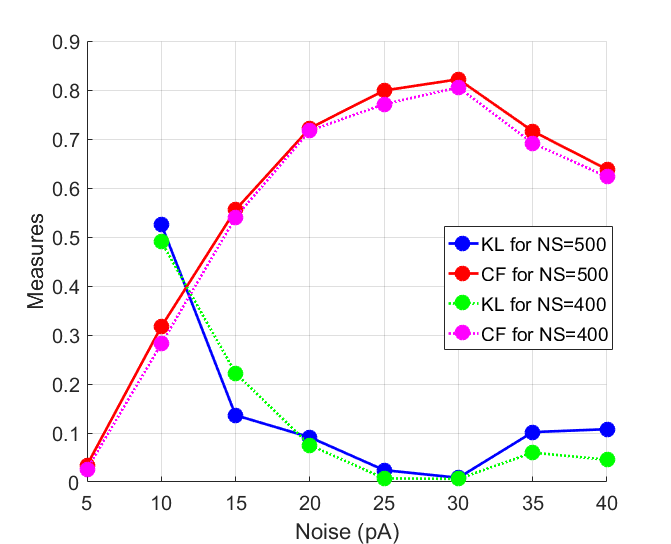


**Figure S3**. Coding fraction and Kullback-Leibler divergence are plotted for FFNs with different levels of noise.


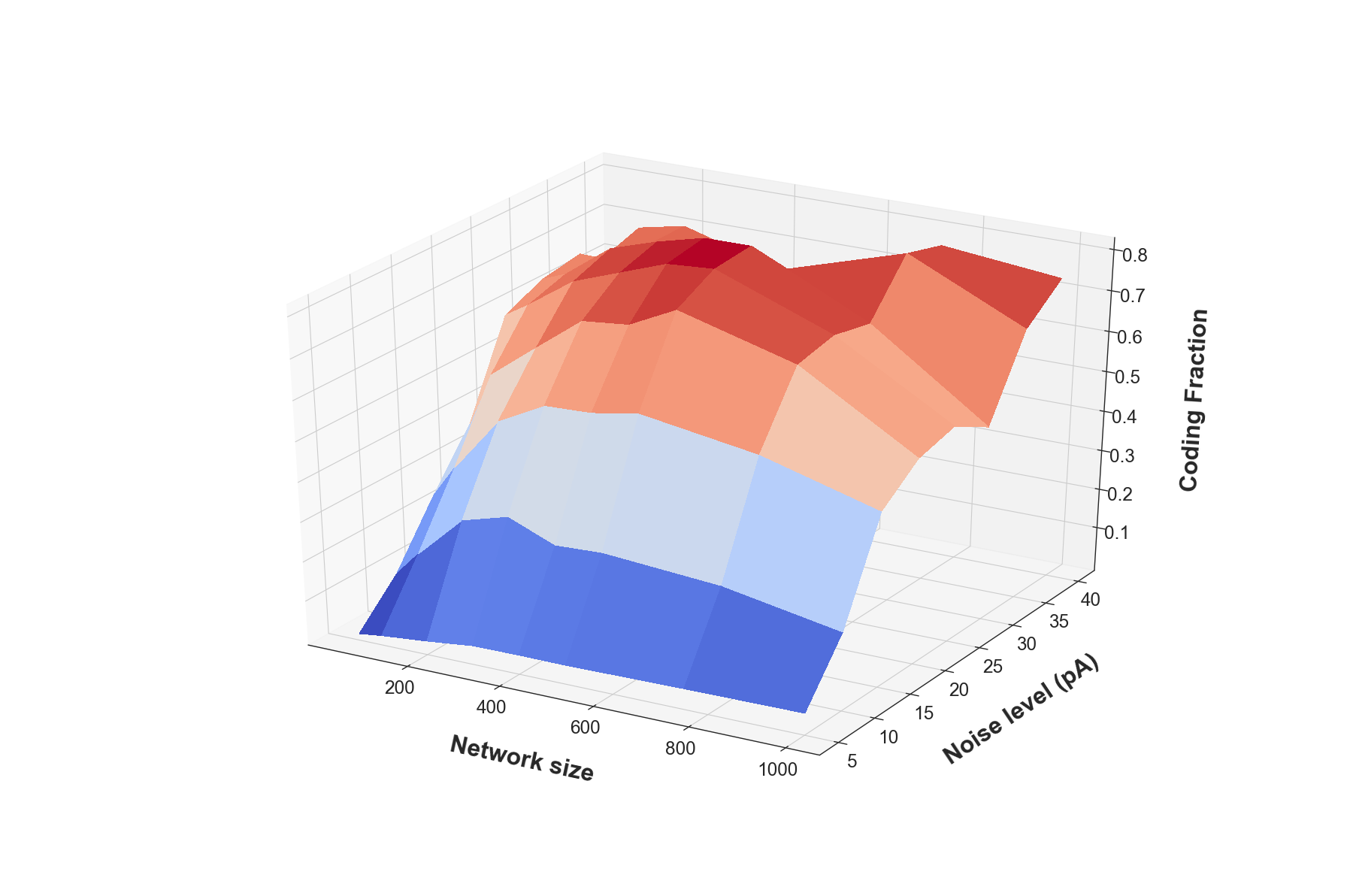


**Figure S4.** 3-D plot of coding fraction as a function of network size and background synaptic noise

**(A)**

**
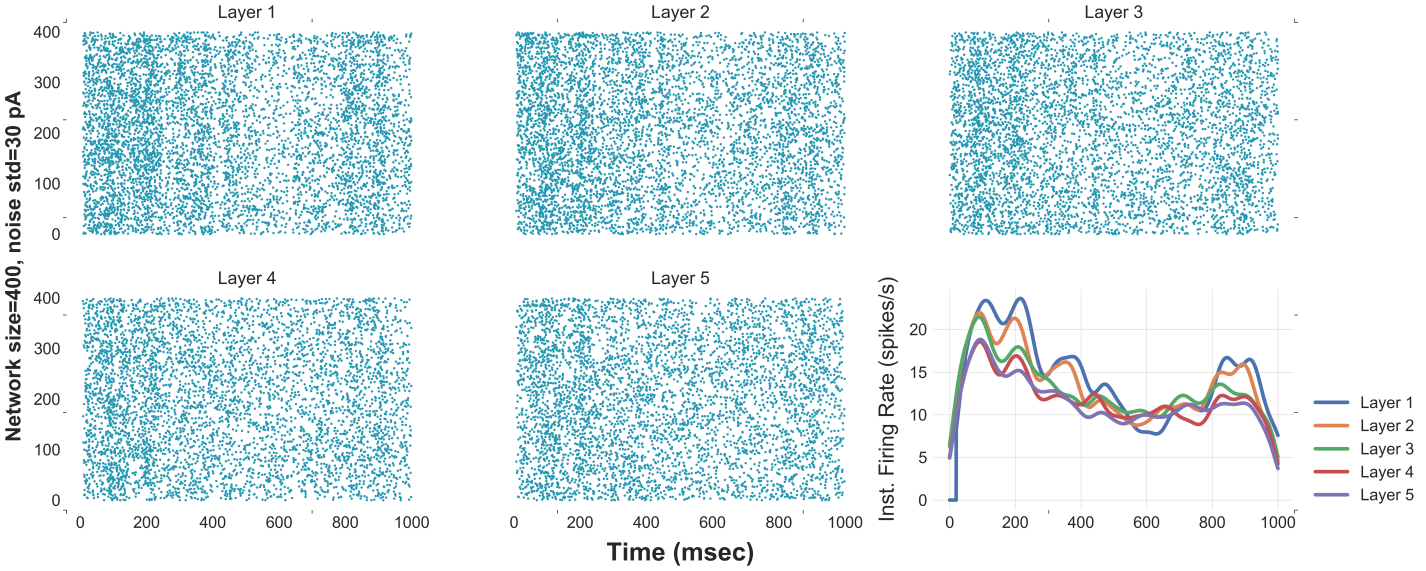
**

**(B)**


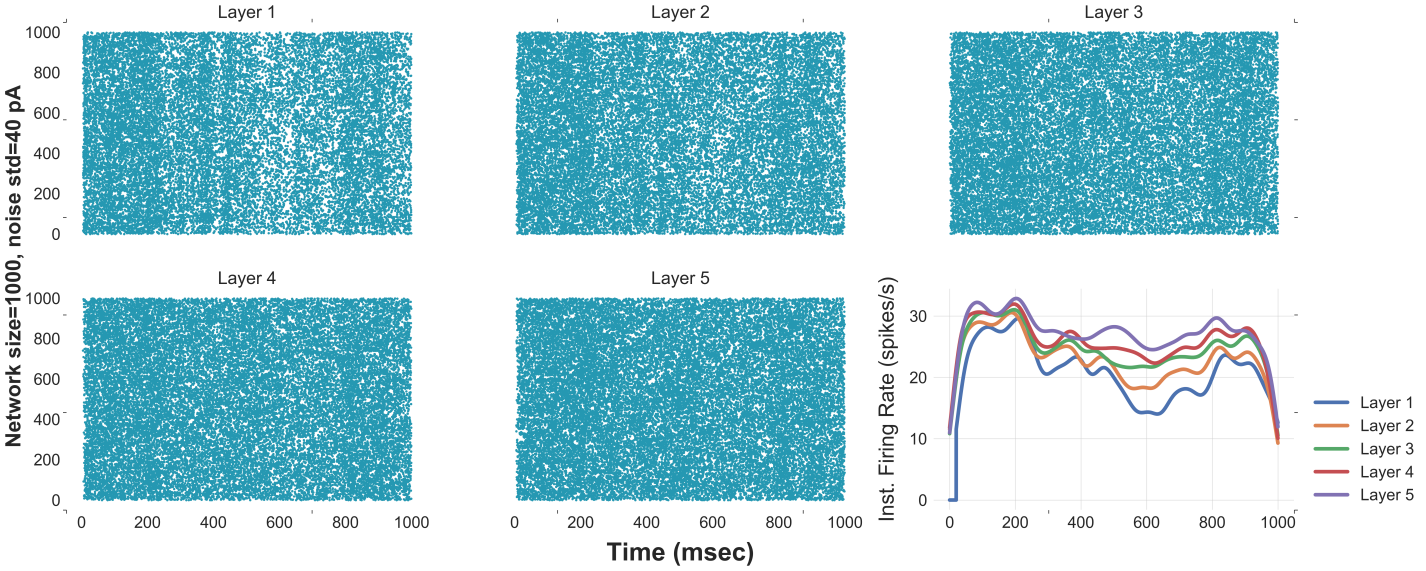


**(C)**


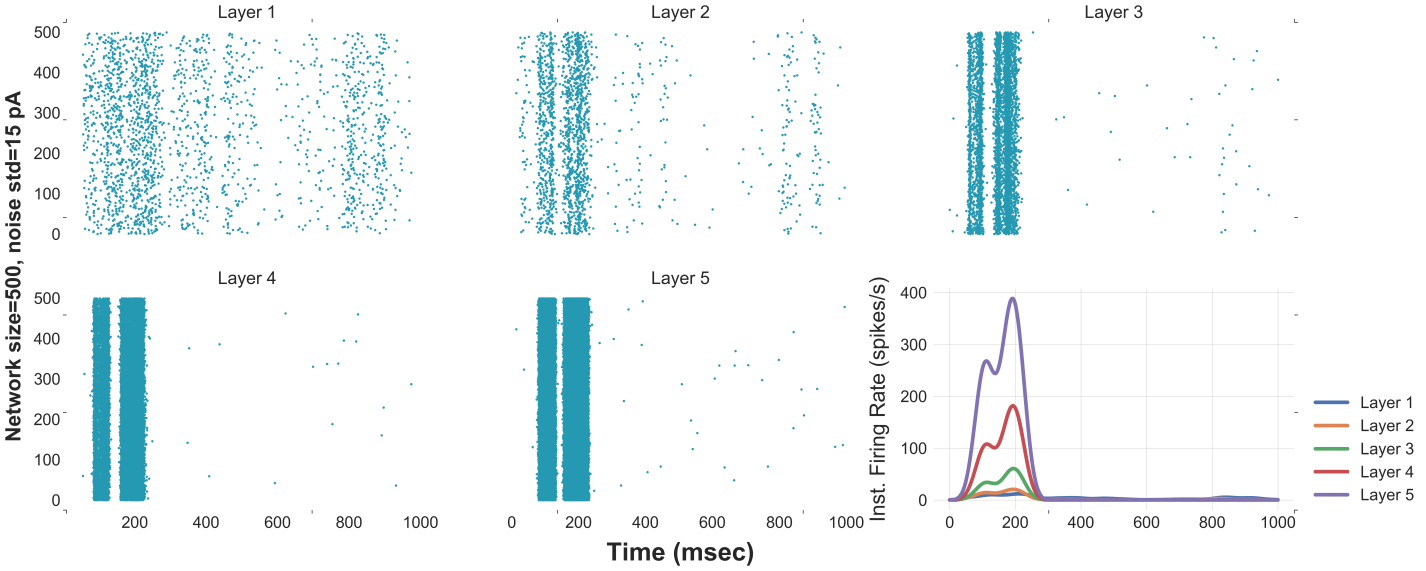


**Figure S5.** Propagation of spikes in 5 layers (raster plot & instantaneous firing rate (Gaussian kernel of width 25 msec)) of FFNs with different pairs of network size and level of synaptic noise. (A) Network size = 400, noise std = 30 pA, (B) Network size = 1000, noise std = 40 pA, and (C) Network size = 500, noise std = 15 pA. Note: the delay between layers is compensated in these curves.

**(A)**


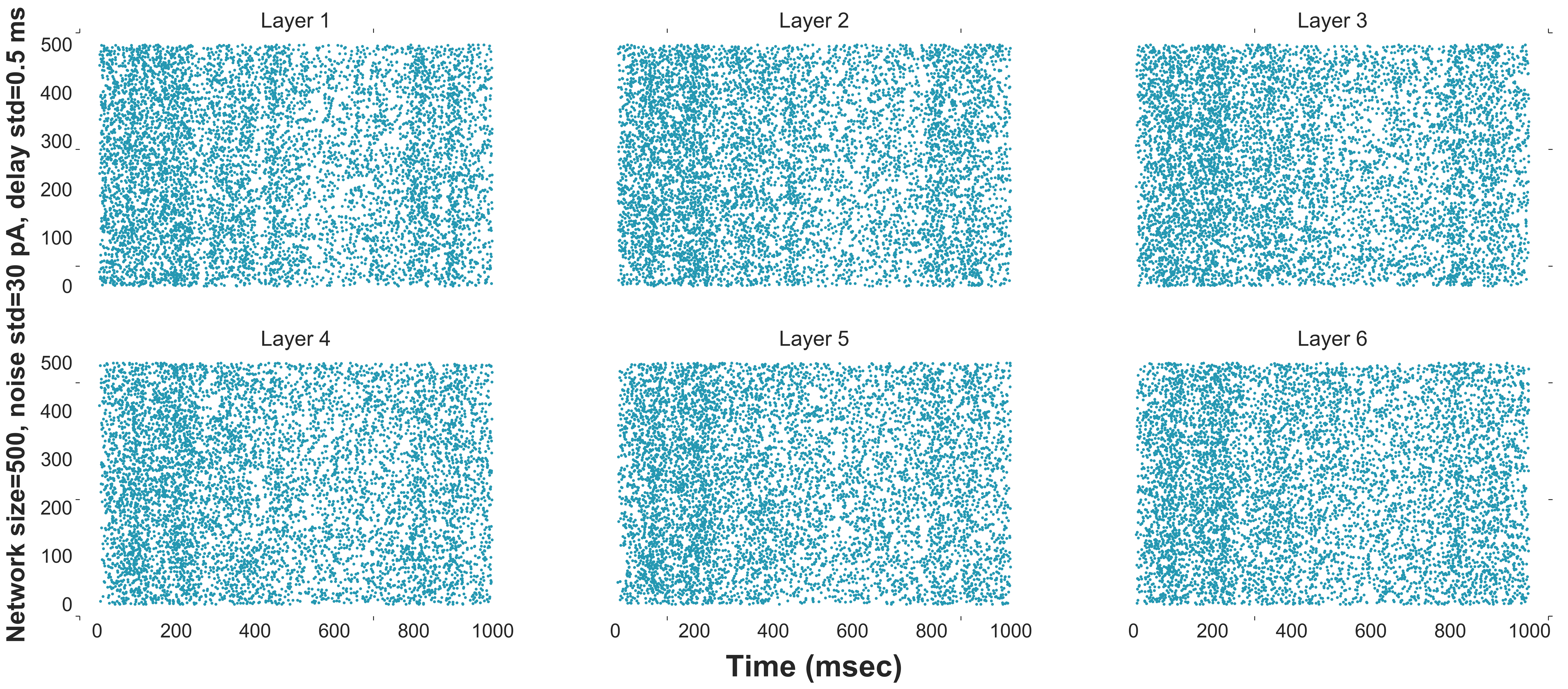


**(B)**


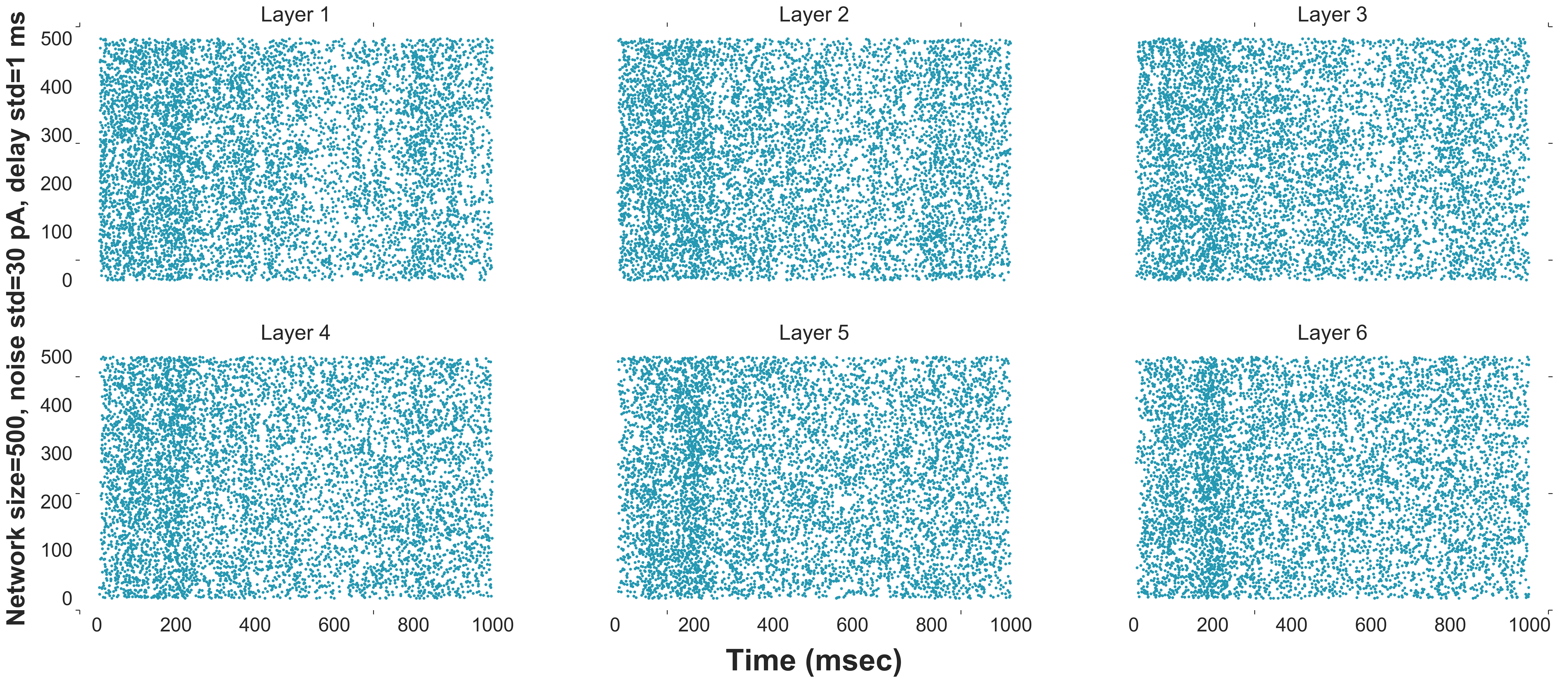


**Figure S6**. Propagation of spikes up to 6 layers with different variabilities of delays. Raster plots are shown for an FFN with N = 500, noise std = 30 pA, and delay std = 0.5 msec (A) and delay std = 1 msec
